# Supplementary material for: Antioxidant and Antiproliferation Activities of Lemon Verbena (Aloysia citrodora): An In Vitro and In Vivo Study
Source: Plants (Basel). 2022 Mar 16;11(6):785. doi: 10.3390/plants11060785 (PMC8951487; doi:10.3390/plants11060785)
Supplement: Supplementary file 1 [file plants-11-00785-s001.zip › plants-1610426-supplementary.pdf]

Figure S1

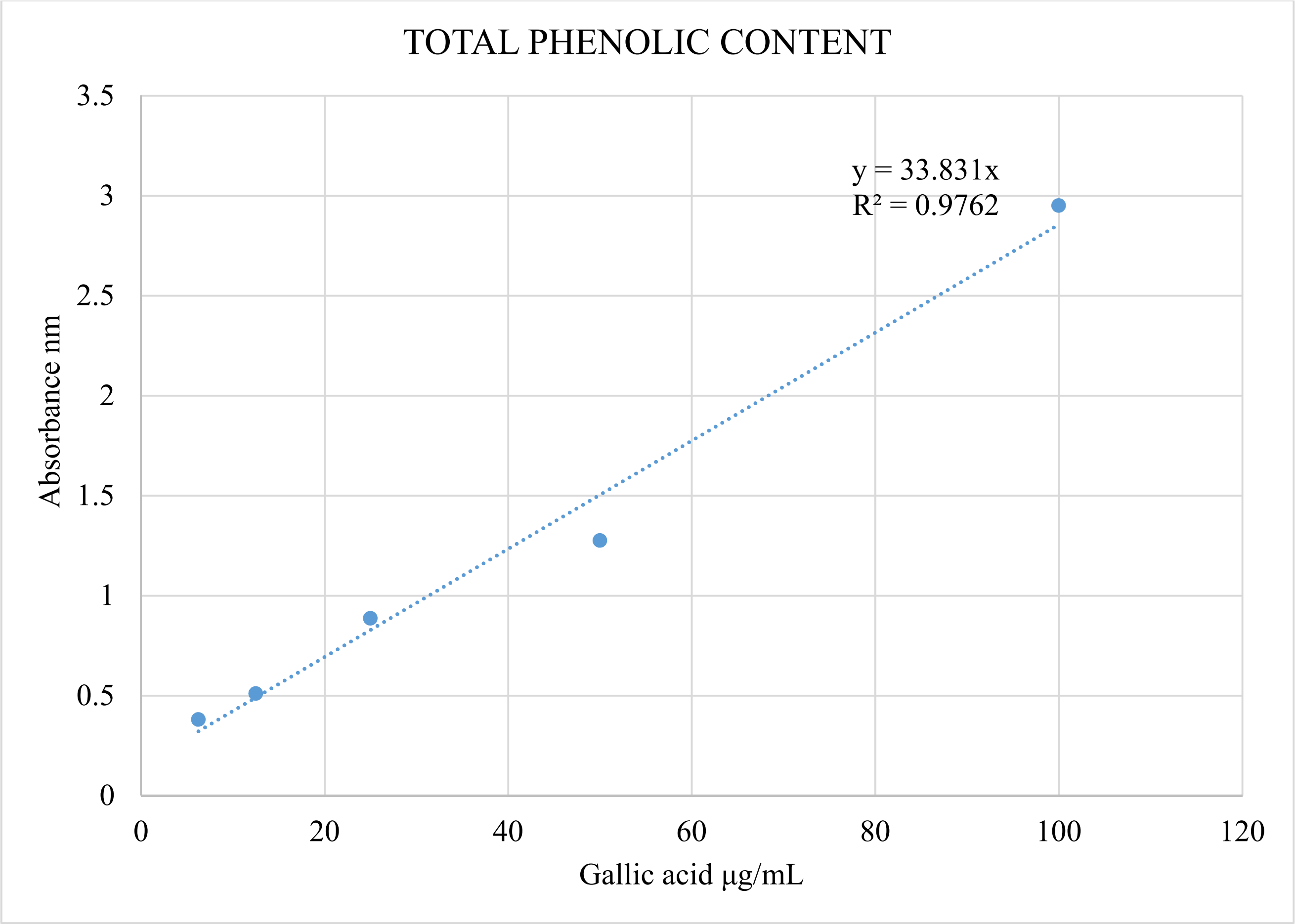

Figure S1: Standard curve for phenolics

Figure S2

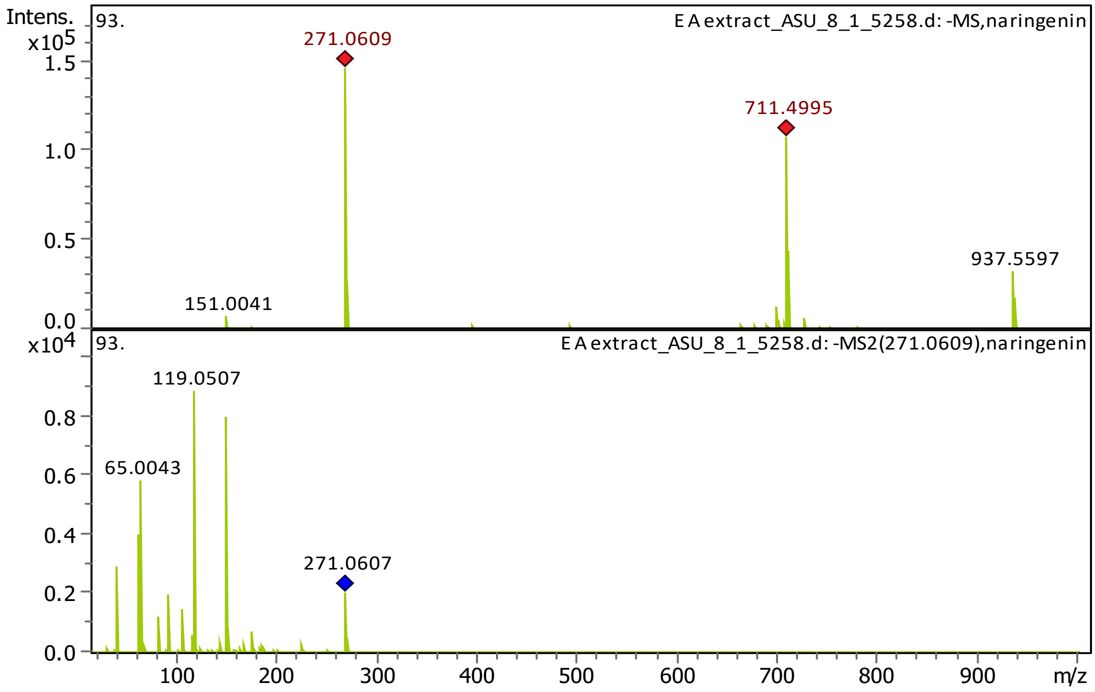
Intens.
x10^5
93.
E A extract_ASU_8_1_5258.d: -MS,naringenin
271.0609
711.4995
937.5597
151.0041
x10^4
93.
E A extract_ASU_8_1_5258.d: -MS2(271.0609),naringenin
119.0507
65.0043
271.0607
m/z

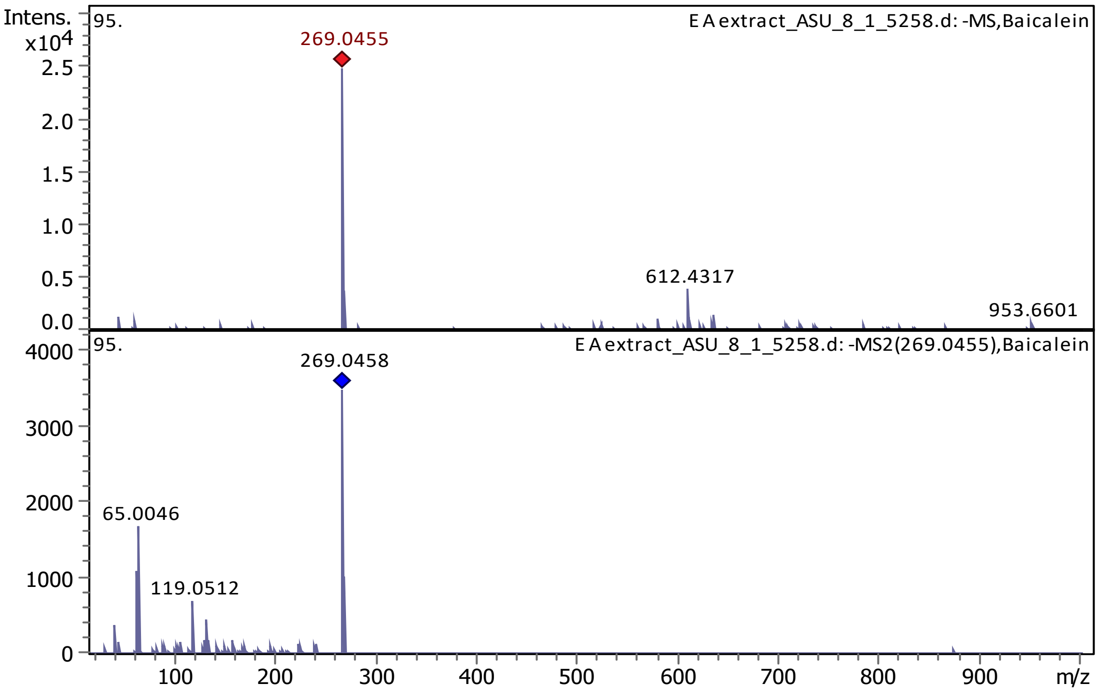
Intens.
x10^4
95.
E A extract_ASU_8_1_5258.d: -MS,Baicalein
269.0455
612.4317
953.6601
95.
E A extract_ASU_8_1_5258.d: -MS2(269.0455),Baicalein
269.0458
65.0046
119.0512
m/z

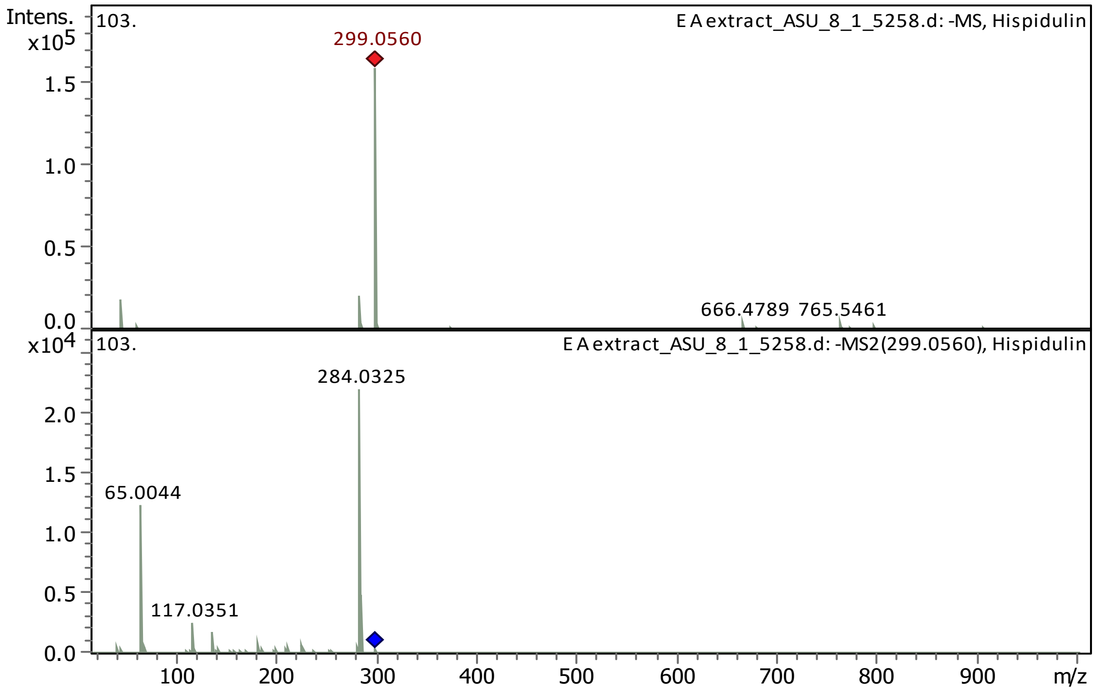
Intens.
x10^5
103.
E A extract_ASU_8_1_5258.d: -MS, Hispidulin
299.0560
666.4789 765.5461
x10^4
103.
E A extract_ASU_8_1_5258.d: -MS2(299.0560), Hispidulin
284.0325
65.0044
117.0351
m/z

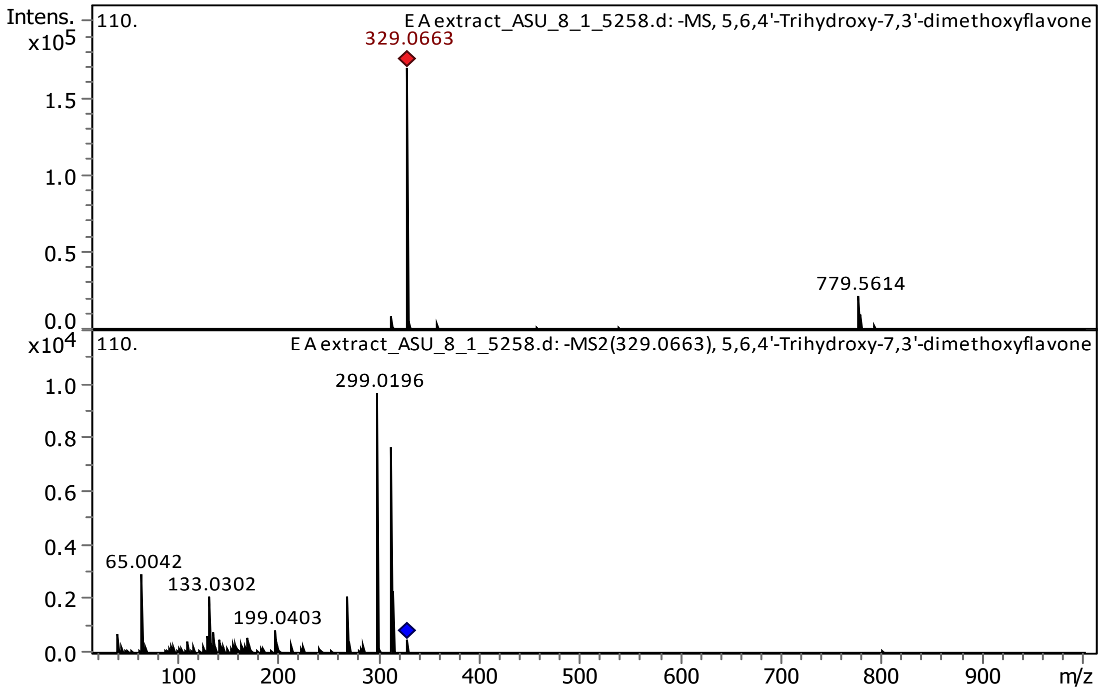
Intens.
x10^5
110.
E A extract_ASU_8_1_5258.d: -MS, 5,6,4'-Trihydroxy-7,3'-dimethoxyflavone
329.0663
779.5614
x10^4
110.
E A extract_ASU_8_1_5258.d: -MS2(329.0663), 5,6,4'-Trihydroxy-7,3'-dimethoxyflavone
299.0196
65.0042
133.0302
199.0403
m/z

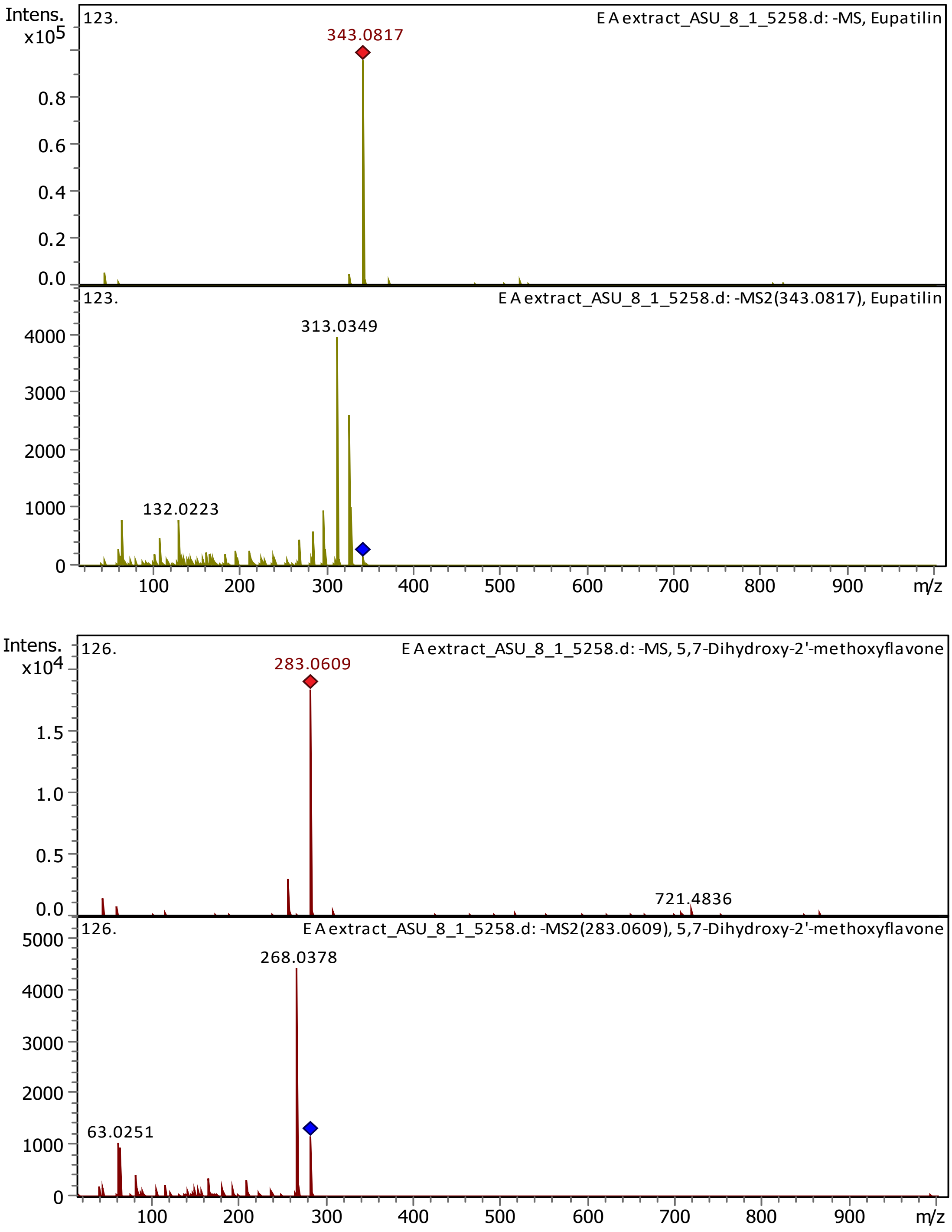

Figure S2: LC-MS Chromatogram obtained from *A. citrodora* ethyl acetate extract

Figure S3

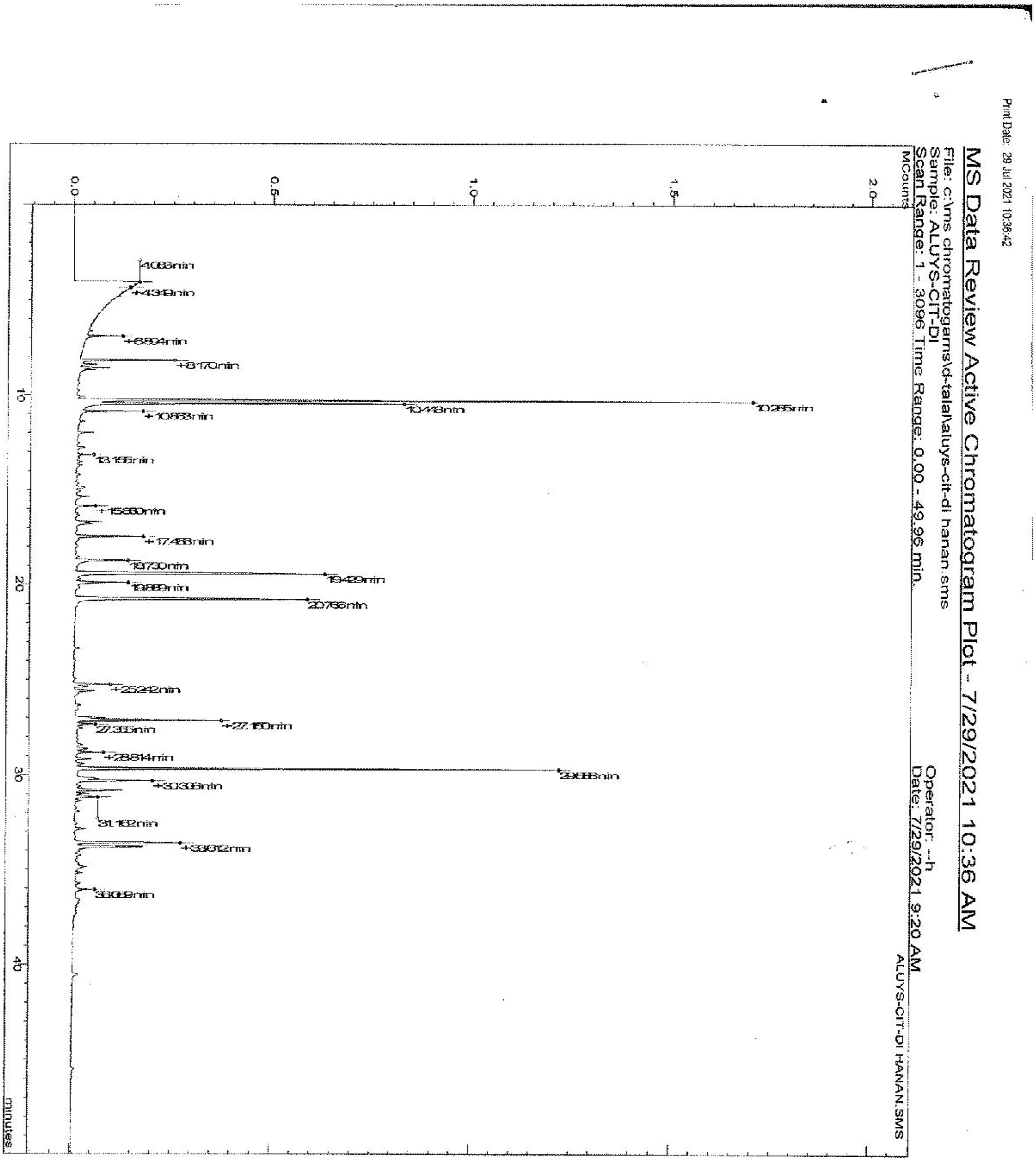

Figure S3: GC-MS analysis of the hydrodistilled oil

Figure S4

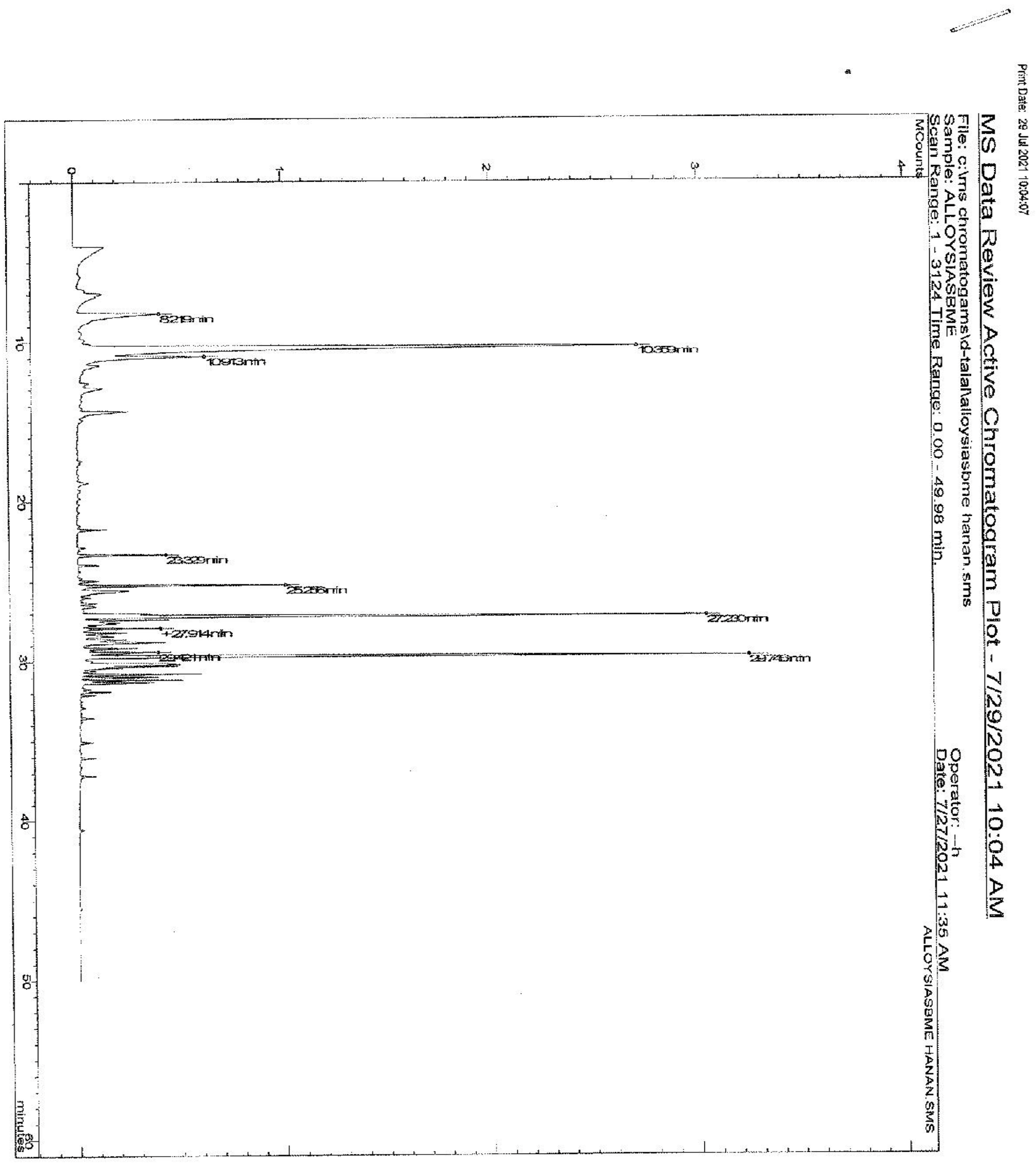

Figure S4: GC-MS analysis of the SPME extracted oil

Figure S5

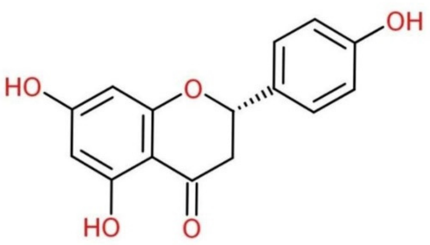

1

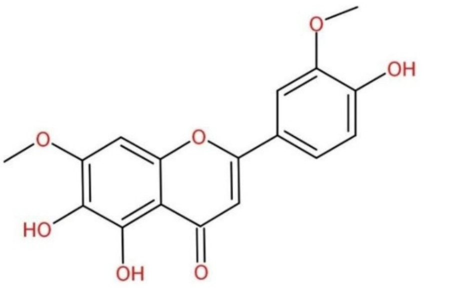

2

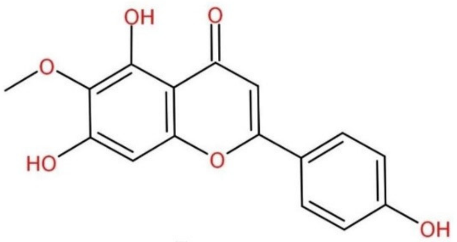

3

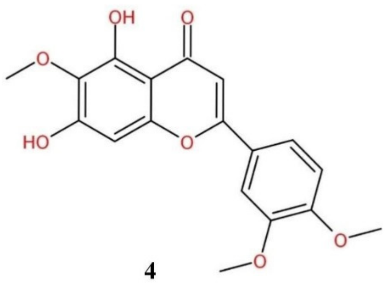

4

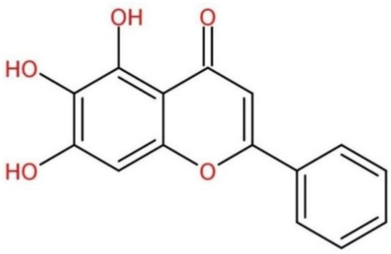

5

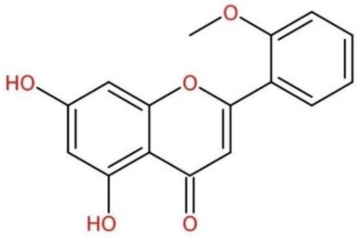

6

Figure S5: Major compounds identified in ethyl acetate extract of A. citrodora using LC-MS. 1: Naringenin, 2: 5-TDMF, 3: Hispidulin, 4: Eupatilin, 5: Baicalein, 6: 2'-Methoxychrysin
